# Research on food loss and waste in the Western Balkans: A systematic review

**DOI:** 10.3389/fnut.2022.983639

**Published:** 2022-09-21

**Authors:** Hamid El Bilali, Sinisa Berjan, Tarek Ben Hassen, Junaid Alam Memon, Željko Vaško, Mohammad S. Allahyari

**Affiliations:** ^1^International Centre for Advanced Mediterranean Agronomic Studies (CIHEAM-Bari), Bari, Italy; ^2^Department of Agroeconomy and Rural Development, Faculty of Agriculture, University of East Sarajevo, East Sarajevo, Bosnia and Herzegovina; ^3^Program of Policy, Planning, and Development, Department of International Affairs, College of Arts and Sciences, Qatar University, Doha, Qatar; ^4^Department of Social Sciences and Liberal Arts, Institute of Business Administration, Karachi, Pakistan; ^5^Faculty of Agriculture, University of Banja Luka, Banja Luka, Bosnia and Herzegovina; ^6^Department of Agricultural Management, Rasht Branch, Islamic Azad University, Rasht, Iran; ^7^Faculty of Economic and Management Sciences, North-West University, Mmabatho, South Africa

**Keywords:** food loss, food waste, sustainable food system, environment, food security, waste management, Balkans

## Abstract

Food losses and waste (FLW) is considered a critical issue in the ongoing debate on the sustainability of agri-food systems. However, the scholarly literature on FLW is still geographically-biased, with more attention devoted to developed countries, even in Europe. In this context, this article analyses the state of research on FLW in the Western Balkan region (viz. Bosnia and Herzegovina, Croatia, Montenegro, North Macedonia, and Serbia). A search performed in October 2021 on the Web of Science database returned 34 documents, and 21 eligible ones were included in the systematic review. The topical analysis of the literature addressed causes of FLW, stages of the food supply chain, extent and magnitude of FLW, FLW and food security, economic and environmental impacts of FLW, and food waste (FW) management strategies. A central finding was the scarcity of data on FW in the Western Balkans. Moreover, the literature focused on FW at the consumer level, while food loss at other stages of the food chain was generally overlooked. There is a lack of comprehensive analyses of the economic and environmental impacts of FLW as well as its implications in terms of food and nutrition security. The quantification of FLW is generally inaccurate and based on estimates and self-reported data. The literature focuses on FW reuse and recycling (e.g., energy, compost) while other management strategies (e.g., reduction/prevention, redistribution) are rarely addressed. However, the results indicated that consumers in the Western Balkans pay attention to the FW issue, especially during the COVID-19 pandemic, which is an encouraging sign that can be exploited in awareness-raising campaigns and education activities. Meanwhile, research on FLW in the Western Balkans is highly needed to fill the identified knowledge gap and provide evidence to policies dealing with the transition to sustainable food systems in the region.

## Introduction

Food loss and waste (FLW) refers to a decrease in food mass at all stages of the food chain (1). It occurs along the whole food chain from harvest to consumption; food loss occurs upstream of the food chain (e.g., production, transport, processing), while food waste takes place downstream of the chain (e.g., retail/distribution and consumption) (1–3). Food wastage is influenced by several behavioral, personal, product, and societal factors (4). FLW generates far-reaching environmental, economic and social impacts (1, 3, 5), is an ethical scandal (6), and represents a serious threat to food and nutrition security worldwide (1, 3, 7). Therefore, food waste management hierarchies present different options for managing waste to reduce its environmental footprints and impacts on food security; they prioritize prevention and redistribution with respect to its use as animal feed, for compost and/or energy production, or its landfill disposal (1, 8). The extent of FLW varies not only among countries but also from one commodity to another; food loss is high in the developing world, while food waste is predominant in developed countries (1–3).

Data on the extent and magnitude of FLW are generally scarce and inaccurate. This is particularly true in developing countries such as those of the Western Balkans. The countries of the Western Balkans generate high amounts of municipal waste (9) - Serbia, 2.46 million tons (0.33 t/capita/year) in 2019; Albania, 1.2 million tons (381 kg/capita/year) in 2019; Bosnia and Herzegovina, 3.25 million tons of waste per year (354 kg/capita); North Macedonia, 456 kg per capita in 2019; and Montenegro, 292.7 thousand tons in 2017. While it is assumed that food represents a significant share of solid municipal waste, accurate data on the share of food waste and its amount are missing.

In a recent systematic review of the literature on agri-food systems worldwide, El Bilali et al. (10) found that the research field is north-biased and dominated by researchers and organizations from developed countries in Europe and North America. It is not clear whether such a statement is true in the case of FLW in general and in the Western Balkans in particular. Nevertheless, as of October 2021, only two reviews indexed in the Web of Science database address FLW in the Western Balkans viz. Petravić-Tominac et al. (11) and Ðurišić-Mladenović et al. (12). However, both reviews do not provide a comprehensive analysis of FLW in the Western Balkans neither geographically nor thematically. Indeed, geographically speaking, the two articles focus on Croatia (11) and Serbia (12). From the thematic viewpoint, they focus on biodiesel production from waste while overlooking many other issues relating to FLW, which is by far a multidisciplinary field. This makes the case and shows the need for a comprehensive review of the existing literature. In this context, the present systematic review analyses the state of research on FLW in the Western Balkans, focusing on Bosnia and Herzegovina, Croatia, Montenegro, North Macedonia, and Serbia. It addresses FLW causes, its extent, and magnitude along the food chain, its economic, environmental, and food security implications as well as FW management strategies.

## Methodology

The article draws upon a systematic review of records indexed in the Web of Science (WoS) database and follows the PRISMA (Preferred Reporting Items for Systematic Reviews and Meta-Analyses) guidelines (13).

### Search source and string

A search was performed on WS on October 16^th^, 2021, using the following search string: (“food loss” OR “food wast^*^”) AND (Bosnia OR Croatia OR Montenegro OR Macedonia OR Serbia OR Balkan OR “South^*^east^*^ *Europe”)*.

### Inclusion and eligibility criteria

For the inclusion in the systematic review, a document had to meet simultaneously three eligibility criteria: (i) the thematic focus (viz. FLW is a central topic in the document); (ii) geographical coverage (viz. the document deals with one or more target countries), and (iii) document type (viz. the document is a journal article, book chapter or conference paper; letters to editors and/or notes were excluded).

### Search strategy and selected documents

**Figure 1:**
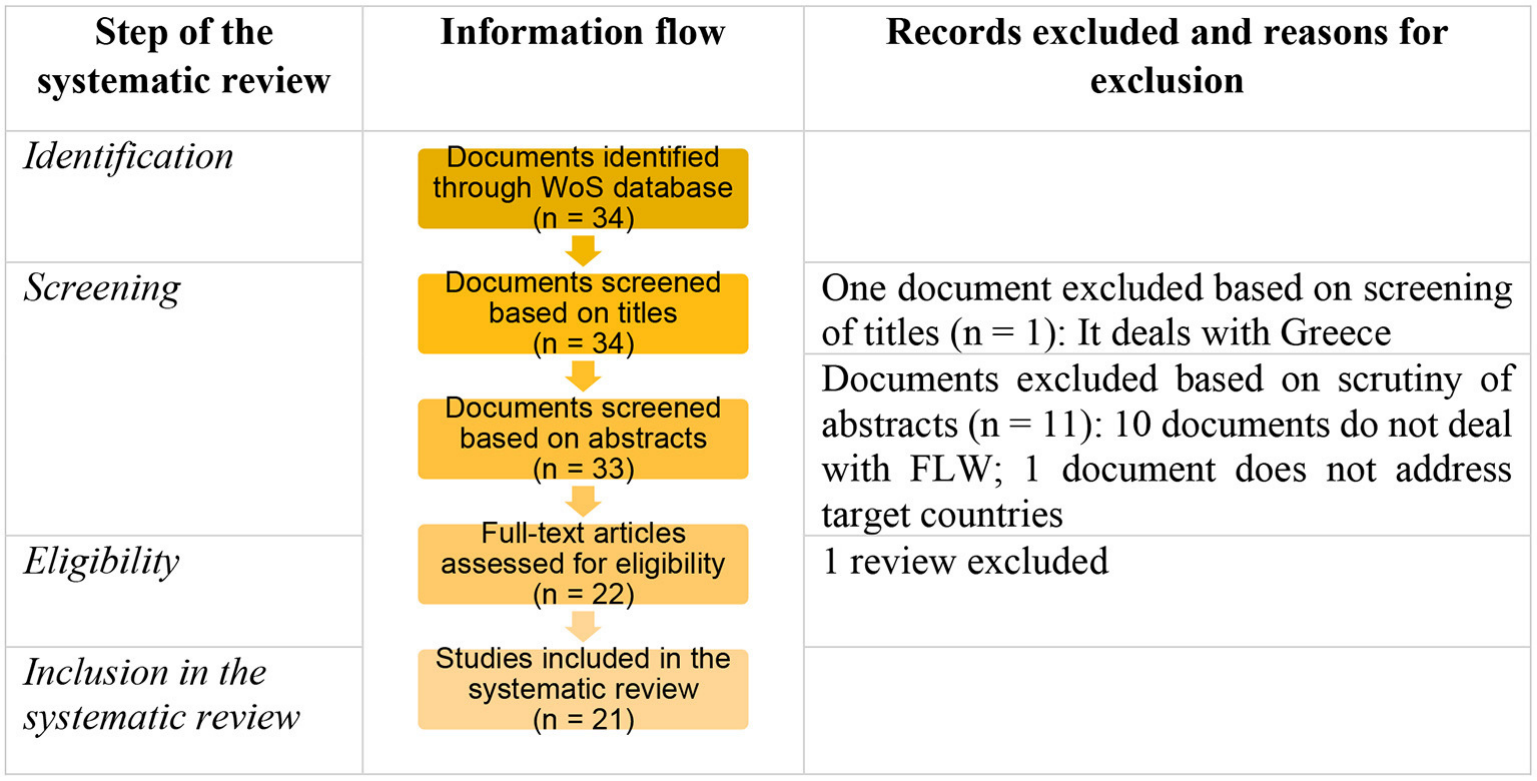
Process of the search and selection of documents.

The initial search on WS returned 34 documents (Figure 1). Following the screening of the titles, one record was excluded as it does not deal with target countries. Additional 11 documents were not considered for further analysis based on scrutiny of abstracts. At this step, documents that address only recycling or reusing animal waste (manure) or “unavoidable food waste” were also excluded. Furthermore, one review was left out. Consequently, 21 documents were considered in the systematic review (Table 1).

**Table 1:** Documents considered in the systematic review.

**Year**	**Number of documents**	**References**
2021	4	Ben Hassen et al. (14), Berjan et al. (15), Guiné et al. (16), Knežević et al. (17)
2020	3	Ilakovac et al. (18), Matek Sarić et al. (19), Voca et al. (20)
2019	7	Berjan et al. (21), Djekic et al. (22), Djekic et al. (23), Djekic et al. (24), Janjic et al. (25), Jotanovic et al. (26), Knezevic et al. (27)
2018	2	Ðurišić-Mladenović et al. (12), Ilakovac et al. (28)
2017	1	Knežević et al. (29), Rutz et al. (30)
2016	1	Rutz et al. (31)
2015	1	Dedinec et al. (32)
2010	1	Njezic et al. (33)

### Content and topical analyses

The content analysis of the selected literature was informed by the methodological approach El Bilali and Ben Hassen (34) adopted in their systematic review on FW in the Gulf Cooperation Council (GCC) countries. Likewise, the limitations of this systematic review are similar to those mentioned by El Bilali and Ben Hassen (34). The topical analysis addressed causes of food wastage, stages of the food supply chain (viz. production, processing, distribution/trade/retail, consumption), extent and magnitude of FW, food wastage and food and nutrition security, economic impacts of food wastage, environmental implications of FW, FW management strategies (e.g., prevention, redistribution, recycling and reuse). The findings from the scholarly literature are analyzed to see whether or not it addresses each specific topic (and to what extent) as well as how the topic was addressed by comparing and discussing data from the different selected documents.

## Results and discussion

### Geography of the research on FLW in the Western Balkans

The systematic review suggests that research on FLW in the Western Balkan countries is relatively recent, with the first WoS-indexed article dating back to 2010 (33). One of the main results of this systematic review is the marginality of research on FLW in the target Western Balkan countries. It is, in fact, rather surprising that only 21 documents dealt with food wastage in the region. However, the analysis of the *research geography* shows that attention devoted by scholars to FLW issue differs across the Western Balkans. Indeed, most analyzed studies were performed in Serbia and Croatia (Table 2). It should be underlined that Croatia is the only country that is a member of the European Union (EU) among the target ones and that might suggest that it is better integrated into the research networks in the EU. Meanwhile, Serbia is the most populous country among the target ones. Nevertheless, there are some multi-country, regional and global studies.

**Table 2:** Geography of the research on FLW in the Western Balkan countries.

**Country or region (number of articles)**	**References**
Bosnia and Herzegovina (2)	Ben Hassen et al. (14), Djekic et al. (23)
Croatia (5)	Ilakovac et al. (28), Ilakovac et al. (18), Knezevic et al. (27), Matek Sarić et al. (19), Voca et al. (20)
Montenegro (1)	Berjan et al. (21)
North Macedonia (1)	Dedinec et al. (32)
Serbia (6)	Berjan et al. (15), Djekic et al. (22), Djekic et al. (24), Ðurišić-Mladenović et al. (12), Janjic et al. (25), Njezic et al. (33)
Western Balkans* (1)	Jotanovic et al. (26)^1^
Europe** (4)	Knežević et al. (29), Knežević et al. (17)^2^, Rutz et al. (31)^3^, Rutz et al. (30)^3^
Global*** (1)	Guiné et al. (16)^4^

^*^This category includes at least two Western Balkan countries.

^**^This category includes at least a European country outside the Western Balkans.

^***^This category includes at least one non-European country.

^1^Former Yugoslavian Republics (Bosnia and Herzegovina, Croatia, Macedonia, Montenegro, Serbia, Slovenia).

^2^Croatia, Poland, Lithuania and Serbia.

^3^Croatia, France, Macedonia and Spain.

^4^Argentina, Brazil, Croatia, Greece, Hungary, Latvia, Lithuania, Poland, Portugal, Serbia, Slovenia, Romania and United States.

### FLW along the food supply chain

The selected articles generally focus on FW at the consumption stage, while little or no attention is paid to the remaining *stages of the food supply chain*, such as production, harvesting, storage, transport and/or processing. Indeed, most of the selected documents address food wastage at the consumer level (e.g., households, restaurants, etc.), while food losses are generally overlooked in the analyzed scholarly literature. Only documents dealing with the valorization, recycling, and reuse of FLW address food losses (12, 30, 31). This is rather surprising as food losses should be more important in the target developing and in-transition countries of the Western Balkans.

### Causes of FLW

There is no article that analyses in a comprehensive way the *causes of FLW* along the whole food chain in the Western Balkan countries. However, some articles address the drivers of food wastage in specific stages of the food chain (mainly consumption) and/or in determined settings (e.g., university canteens, restaurants, households). Voca et al. (20) argue that plate waste in restaurants in Zagreb (Croatia) is affected not only by the portion size (leftover and waste increase with portion size) but also the type of meals (vegan meals are less wasted than dishes including food of animal origin). Meanwhile, Janjic et al. (25) enumerate too long storage, overconsumption, and improper preparation, among the reasons leading Serbian households to throw away significant amounts of food. Ilakovac et al. (28) report that the main culprits in household food wastage in Croatia are preparing excessive amounts of food for meals and purchasing an excessive quantity of food. Djekic et al. (23) found that the highest-ranked reason behind household FW in Bosnia and Herzegovina is linked to plate leftovers. In Serbia, Djekic et al. (22) found two main reasons for throwing away food: expiration dates and rotten smells and tastes. Meanwhile, Knezevic et al. (27) found that attitude toward food wastage among university students in Croatia is affected by the awareness of FW problem, concerns about environmental and economic impacts of FW, health concerns, and awareness and concerns about the expiration date.

### Extent of FLW

The selected documents do not include any comprehensive *quantification of FLW* in the Western Balkan countries. Furthermore, most of the few presented figures on household FW quantities are based on estimates and self-reported data and do not make any distinction between edible (cf. avoidable FW) and inedible (cf. unavoidable FW) parts of food (Table 3). Some scholars also analyzed the *most wasted types of food*. Djekic et al. (22) argue that bread and bakery products are the most discarded types of food in Serbia. Likewise, Berjan et al. (21) found bakery products are the most wasted foods in Montenegro. Also, Njezic et al. (33) show a significant amount of leftover bread in Serbia.

**Table 3:** Quantification of FLW in the Western Balkan countries.

**Document**	**Country**	**Type of FLW**	**FLW quantity**
Ilakovac et al. (18)	Croatia	Household food waste	75 kg/person/year
Djekic et al. (23)	Bosnia and Herzegovina	Household food waste	2.8 kg/household/week
Djekic et al. (22)	Serbia	Household food waste	1.54 kg/household/week
Djekic et al. (24)	Serbia	Household food waste from animal origin	200 g/household/week (11.3 kg household/year)

Most studies report that food wastage is low in the Western Balkans, with respect to other more developed European countries, and highlight that consumers in the region have a positive attitude and are willing to reduce food wastage (14, 15, 19, 21–23, 25). However, Guiné et al. (16) explore motivations influencing people's eating habits toward sustainability in 13 countries (viz. Argentina, Brazil, Croatia, Greece, Hungary, Latvia, Lithuania, Poland, Portugal, Serbia, Slovenia, Romania and the United States) and argue that “*Although people avoid food waste at home, the awareness for the waste at restaurants still needs to be improved*.”

### FLW during the COVID-19 pandemic

More recently, some studies analyzed the effects of the *COVID-19 pandemic*, and the related containment measures, on FLW in some Western Balkan countries such as Bosnia and Herzegovina (14) and Serbia (15). Both studies point out that the pandemic affected food-related practices and behaviors, including food wastage, but the findings are rather mixed; for instance, Ben Hassen et al. (14) report that the pandemic improved the awareness of Bosnians toward food with a decrease in FW while Berjan et al. (15) found that household food wastage increased in Serbia during the COVID-19 pandemic.

### Implications of FLW in terms of food security

The selected articles do not address the relationship between FLW and food security; this is probably because food security is not considered an issue in the Western Balkans but denotes, anyway, a gap in the research field. However, many authors refer to food security to justify reducing FLW in the Western Balkans. For instance, the purpose mission of social supermarkets, promoted in Europe during the 2008–2014 economic crisis, is to decrease FW to improve access to food by socially marginalized groups (17), thus combatting food poverty. Knežević et al. (29) point out that various social initiatives, based on cross-sector cooperation in food distribution, try to simultaneously address the problems of food poverty and FW in Croatia and other countries in the EU. Ðurišić-Mladenović et al. (12) warn that the use of edible vegetable oils as a potential feedstock for biodiesel in Serbia cannot be considered as a long-term, sustainable choice due to the associated food vs. fuel debate i.e., trade-offs between biofuel production and food security.

### Economic and environmental impacts of FLW

The literature included in the systematic review suggests that no article addresses the broad *economic impacts of FLW*. Furthermore, the 21 selected articles do not examine neither the impacts of FLW on the prices of agri-food products in the Western Balkan countries nor how such price changes affect consumers and producers, which are two recurring topics in the literature on the economic implications of FLW (35, 36). However, some articles estimate the financial value of food lost and/or wasted in different settings as well as the economic benefits of recycling or reusing FLW. Berjan et al. (21) argue that the monthly economic value of FW among Montenegrin households is 5–25 Euro. Ilakovac et al. (28) report that about two-thirds of the interviewed Croatian households consider FW a financial loss. Likewise, Knezevic et al. (27) argue that concerns about the economic aspects of FW affect the awareness of university students toward food wastage in Croatia. Interestingly, Djekic et al. (24) study shows that Serbian consumers are more aware of FW's negative social and environmental impacts than of its associated negative economic dimensions.

Further, no article among the selected ones comprehensively analyses the *environmental footprints of FLW* in the Western Balkan countries. Furthermore, no article dealt with neither the externalities of FLW (e.g., water pollution, ecosystem disturbance, biodiversity loss, deforestation) nor the relationship between FLW and climate change. Nevertheless, Djekic et al. (23) report that food wastage contributes to global warming (cf. climate change), acidification, and eutrophication. Likewise, Djekic et al. (22) suggest that “*The global warming impact of food waste and food packaging waste was quantified around 3.46 kg CO*_2_*e/household a week and 0.16 kg CO*_2_*e/household a week, respectively*” (p. 44). Djekic et al. (24) argue that animal origin FW contributes significantly to CO_2_ emissions in Serbia. A study carried out by Guiné et al. (16) shows that environmental motivations that affect people's food habits in many countries (including Croatia and Serbia) relate, among others, to biodiversity preservation, natural resources and energy saving, and industrial pollution reduction.

Meanwhile, Ilakovac et al. (28) found that about two-thirds of the Croatian households consider FW bad for the environment. Matek Sarić et al. (19) found that many different socio-demographic factors influence environmentally-concerned food choices in Croatia, such as age, gender, education level, and marital status. Knezevic et al. (27) point out that concern about the environmental impacts is one factor affecting the attitude of Croatian university students toward FW. *Environmental awareness* about FW changes from a country to another even within the Western Balkan region. In this regard, in their analysis of consumers' environmental management of FW of the former Yugoslavian Republics, Jotanovic et al. (26) found that there are significant differences between countries; respondents from Slovenia (that is a member of EU since 2004) show a significantly higher level of ecological disposal of FW, while respondents from Macedonia are less prone to dispose of FW in an ecological manner in comparison to Bosnian and Croatian ones.

### FLW management

The considered articles mention different *food waste recovery strategies* ranging from FLW prevention and/or reduction to FLW recycling and reuse (e.g., animal feed, composting, industrial uses such as the production of biogas/biofuel) through food surplus redistribution (donation to food banks, social supermarkets, etc.), which are strategies contemplated in the food waste management hierarchy (37, 38). Ilakovac et al. (28) found that only a small share of the Croatian households feed food leftovers to dogs or cats while many still simply discard leftovers in the bin. Njezic et al. (33) suggest that leftover bread represents an environmental problem in Serbia (especially when landfilled), but also a potentially valuable raw material as animal feed. Ðurišić-Mladenović et al. (12) analyze the biodiesel production potential of Serbia and highlight that besides conventional biodiesel sources (e.g., oilseed crops) many other sources can be used, including waste cooking oils as well as oils from non-edible agro-food wastes. Rutz et al. (30, 31) show the potential of biomethane production and its use in transport by using segregated FW (cf. catering sector, households, food, and beverage industry) as feedstock in four European cities [Zagreb (Croatia), Skopje (Macedonia), Malaga (Spain), and Paris (France)] and highlight the multiple environmental benefits of source-separated waste collection and conversion into biomethane in comparison to other waste treatment methods (e.g., landfilling, incineration, composting). Dedinec et al. (32) use the Greenhouse Gas Costing Model (GACMO) and highlight that sustainable management of waste (including FW) can contribute to climate change mitigation measures in developing countries such as North Macedonia. Indeed, the use of the appropriate waste treatment methods (e.g., mechanical biological treatment–MBT–with composting, MBT with anaerobic digesters for energy production) can reduce both GHG emissions and waste management costs (32). Different socio-demographic factors affect the quantities of FW produced and food wastage prevention potential. Ilakovac et al. (18) report that in Croatia the respondent age positively affected FW prevention, while the household income level and size (especially the number of children and teens under 18) had an adverse effect. Knežević et al. (17) analyse the perception of consumers in Central Eastern Europe (viz. Croatia, Poland, Lithuania, and Serbia) toward social supermarkets and put that there is a positive attitude regarding the role of social supermarkets FW reduction.

## Conclusion

To the best of our knowledge, this is the first systematic review on FLW in the Western Balkans. The relevance of the present systematic review lies not only in informing the actors of the food supply chain (e.g., policymakers, scientists, etc.) on the landscape of research on FLW in the region but also in synthesizing available data and findings, highlighting the existing knowledge gaps, and identifying research needs in the field. The literature review clearly shows that research on FLW is still marginal in the Western Balkans. Furthermore, the analysis of the available literature shows that the research focuses on FW at the consumer level, while food losses are often overlooked. The existing research gaps also include causes and drivers of FLW, extent, and quantification of FLW along the whole food supply chain, implications of food wastage for food security, and economic impacts and environmental footprints of FLW. Future research should also pay more attention to the type of wasted food (cf. avoidable vs. unavoidable FW) as it determines not only environmental and economic impacts but also appropriate FW management strategies (viz. prevention, redistribution, recycling, and reuse). While FLW prevention and reduction should be given priority, it is also vital to emphasize strategies relating to the reuse and recycling of FW (cf. composting, energy production, industrial use). As the causes of FLW are different, also solutions depend on the food supply chain stage and differ among countries and regions. In this respect, the integration of FW reduction into policy is crucial. Moreover, the efficiency of FW reduction solutions depends on the participation and inclusiveness of a wide range of food supply chain stakeholders. The review highlights the lack of accurate statistics and data about food wastage in the countries of the Western Balkans, and research should help provide data on the magnitude and extent of FW in the region. Given the magnitude of FLW in Western Balkan countries and the obvious knowledge gaps, further research is needed to inform future policy and action. The generation of new data through research is indispensable for designing effective and efficient policies and strategies toward addressing FLW issues in the region. The results indicate that consumers in the Western Balkans pay increasing attention to the FW issue, which is an encouraging sign that can be exploited in other awareness-raising campaigns and education activities. In order to be effective in reducing and/or preventing food wastage, these activities should consider their FW habits, starting from procurement planning through food storage and meals preparation. The reduction of FLW is paramount for food and nutrition security, climate change mitigation, and sustainable management of natural resources in the broader context of sustainable food systems. Therefore, curbing FLW is crucial to achieving the Sustainable Development Goals (SDGs) in the Western Balkans.

## Author contributions

HE, SB, and TB: conceptualization, methodology, and writing—review and editing. HE and SB: formal analysis and project administration. MA: data curation. HE, SB, TB, JM, ŽV, and MA: writing—original draft preparation. All authors have read and agreed to the published version of the manuscript.

## Funding

The publication of this article was funded by the Qatar National Library.

## Conflict of interest

The authors declare that the research was conducted in the absence of any commercial or financial relationships that could be construed as a potential conflict of interest.

## Publisher's note

All claims expressed in this article are solely those of the authors and do not necessarily represent those of their affiliated organizations, or those of the publisher, the editors and the reviewers. Any product that may be evaluated in this article, or claim that may be made by its manufacturer, is not guaranteed or endorsed by the publisher.
